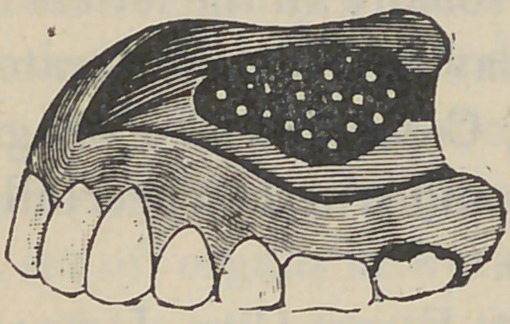# A Plate Retainer

**Published:** 1894-03

**Authors:** Wm. H. Steele

**Affiliations:** Forest City, Iowa


### A Plate Retainer.

BY WM. H. STEELE, FOREST CITY, IOWA.

The following plan has been in use in my practice for several years, and has proved a perfect success in overcoming those difficult cases, where we have a *soft alveolar ridge* in connection with a very firm, hard palate. Take the impression with Teague's compound, and make cast as usual; after the cast is hard enough for the base plate, examine the mouth carefully and locate the margins of the hard palate. Cut a pattern from heavy paper, the proper size and shape to cover this part of the mouth; now place this pattern on a piece of tin of the proper thickness, and cut out a fac-simile of the paper; fit this on the plaster cast *exactly* in the same position that the pattern was fitted to in the mouth; press it firmly to place, and fasten with cement, or a couple of pins. Now make the base plate, and mount in the articulator as usual; in grinding up be careful to get a *perfect* articulation; invest as usual, remove the wax and pack with best maroon or black rubber until there is just enough for the plate. Then cut a piece of the cloth, which comes with the rubber, large enough to fully cover the cast, and lay over it in order that the flask may be easily opened; bring the flask *entirely* together. Open and remove the cloth and the tin pattern from the cast. Fold sheets of black soft *palate* rubber, until it is one half thicker than the tin; lay the pattern on this and cut the rubber same shape and size. Place this piece of rubber in the depression made in the rubber by the tin pattern. Close and vulcanize as usual. When the plate is all finished, pit the surface all over as shown in the cut, with a number seven round bur, while doing this, the rubber and bur must be kept wet. The soft rubber should follow the hard ridge entirely back to the heel of plate as shown in cut. The thickness of the soft rubber should be governed by the variation in difference between the hard and soft tissues, and often the tin pattern should be thinned with a file in some parts before fastening to cast, in order to make the soft rubber thickest in the hardest part of the arch.

## Figures and Tables

**Figure f1:**